# Unraveling the Role of RSPRY1 in TGF-β Pathway Dysregulation: Insights into the Pathogenesis of Spondyloepimetaphyseal Dysplasia

**DOI:** 10.3390/ijms26031134

**Published:** 2025-01-28

**Authors:** Gozde Imren, Beren Karaosmanoglu, Bihter Muratoglu, Cansu Ozdemir, Gulen Eda Utine, Pelin Ozlem Simsek-Kiper, Ekim Z. Taskiran

**Affiliations:** 1 Department of Medical Genetics, Faculty of Medicine, Hacettepe University, 06100 Ankara, Türkiye; gimren@hacettepe.edu.tr (G.I.); berenk@hacettepe.edu.tr (B.K.); 2 Department of Medical and Surgical Research, Institute of Health Sciences, Hacettepe University, 06100 Ankara, Türkiye; 3 Department of Stem Cell Sciences, Institute of Health Sciences, Hacettepe University, 06100 Ankara, Türkiye; 4 Center for Stem Cell Research and Development (PEDI-STEM), Hacettepe University, 06100 Ankara, Türkiye; 5 Department of Pediatric Genetics, Faculty of Medicine, Hacettepe University, 06100 Ankara, Türkiye

**Keywords:** RSPRY1, TGF-β signaling, extracellular matrix, spondyloepimetaphyseal dysplasia, genome editing

## Abstract

Skeletal dysplasias, characterized by bone, cartilage, and connective tissue abnormalities, often arise due to disruptions in extracellular matrix (ECM) dynamics and growth factor-dependent signaling pathways. RSPRY1, a secreted protein with RING and SPRY domains, has been implicated in bone development, yet its exact role remains to be determined. *RSPRY1* gene mutations are associated with spondyloepimetaphyseal dysplasia (SEMD), a rare skeletal disorder characterized by severe epiphyseal and metaphyseal deformities. This study aimed to determine the molecular and cellular mechanisms by which RSPRY1 deficiency affects skeletal homeostasis. Transcriptome analysis of fibroblasts from patients with homozygous *RSPRY1* mutations showed there was significant enrichment of transforming growth factor beta (TGF-β) signaling and ECM-related pathways. Functional wound healing assays showed that *RSPRY1* knockout fibroblasts exhibited enhanced motility, a phenotype that was abrogated in *RSPRY1* + *SMAD3* double knockout fibroblasts, highlighting the SMAD3-dependence of RSPRY1′s effects. The observed limited response to exogenous TGF-β in RSPRY1-deficient cells indicated that there was constitutive pathway activation. These findings show that RSPRY1 is a critical regulator of TGF-β signaling in ECM dynamics and cell motility, contributing to the pathophysiology of SEMD. An improvement in our understanding of the molecular roles of RSPRY1 might yield novel therapeutic strategies that target TGF-β signaling in patients with SEMD and other skeletal dysplasias.

## 1. Introduction

The *RSPRY1* gene is located on chromosome 16q13 and encodes a 576-amino acid protein containing two functional domains: the RING (really interesting new gene) finger domain, known for its role in protein–protein interactions (PPIs) and ubiquitination processes, and the SPRY (SPla and the RYanodine receptor) domain, which facilitates protein binding and cellular signaling [1]. The combination of these domains positions RSPRY1 as a potential regulator of extracellular processes, particularly given its classification as a secreted protein in such databases as BioGRID and UniProt [2,3]. Whereas the precise biological function of RSPRY1 remains unknown, biallelic *RSPRY1* mutations are known to be linked to spondyloepimetaphyseal dysplasia Faden-Alkuraya type (SEMDFA, OMIM #616723), a progressive genetic skeletal disorder characterized by vertebral defects, mild scoliosis, epiphyseal and metaphyseal abnormalities, short stature, facial dysmorphism, short fourth metatarsals, intellectual disability, and in some cases craniosynostosis [4,5,6]. Faden et al. observed a strong expression of RSPRY1 in osteoblasts and osteocytes during the mid-to-late embryonic stages in mouse models, particularly in developing endochondral bones and skeletal tissues [4,5,6]. These findings collectively highlight the essential role of RSPRY1 in skeletal development, cellular homeostasis, and tissue formation; however, the precise molecular mechanisms by which RSPRY1 deficiency affects cellular processes in the skeletal system have yet to be elucidated.

The skeletal system is the human body’s fundamental structural and functional component. It is regulated by complex cellular and molecular processes that govern its development, homeostasis, and remodeling. Among these processes, extracellular matrix (ECM) dynamics and growth factor-dependent signaling pathways play a central role [7]. The ECM provides mechanical support and functions as a regulatory microenvironment, controlling cell–ECM interactions, migration, and differentiation via signaling molecules [8].

The complexity of ECM regulation and skeletal development underscores the critical role of genetic factors in these processes. Genetic mutations can disrupt cellular differentiation pathways, ECM production, or growth factor signaling, leading to imbalances in skeletal homeostasis [9]. Such disruptions underlie many skeletal dysplasias, a diverse group of disorders characterized by abnormal bone, joint, and cartilage development [10]. Transforming growth factor beta (TGF-β) signaling is a critical key regulator of skeletal development [11,12]. TGF-β regulates cell proliferation and differentiation and ECM synthesis, particularly during cartilage and bone formation [12,13]. TGF-β ensures proper cell–ECM interactions during bone development via context-dependently modulating cellular responses [14]. Dysregulation of TGF-β signaling is commonly associated with skeletal abnormalities and pathological conditions [11,14].

Despite recognition of the role TGF-β signaling plays in skeletal homeostasis, the upstream modulators of this pathway remain poorly defined. The present study hypothesized that RSPRY1, a key player in ECM regulation and growth factor signaling, is a critical upstream regulator of TGF-β signaling. RSPRY1 is thought to act as a molecular mediator that regulates cell–ECM interactions and ECM dynamics that are essential for skeletal homeostasis. In particular, its potential interaction with the TGF-β signaling pathway—a central regulator of skeletal tissue development and homeostasis—has not been previously observed.

The present study is, to the best of our knowledge, the first to provide direct evidence that RSPRY1 deficiency leads to overactivation of the TGF-β signaling pathway, revealing a novel molecular mechanism underlying the pathogenesis of spondyloepimetaphyseal dysplasia (SEMD). Transcriptomic analysis showed patient-derived fibroblasts’ differentially expressed genes (DEGs) notably enriched TGF-β signaling-related pathways. Furthermore, the present study’s analyses identified disease-associated genes that interact with RSPRY1 and the TGF-β signaling pathway. These findings led to the hypothesis that RSPRY1 deficiency activates TGF-β signaling, contributing to ECM remodeling defects and skeletal deformities.

To test this hypothesis, *RSPRY1* knockout (KO), *SMAD3* KO, and *RSPRY1* + *SMAD3* double KO fibroblasts were generated using Cas9/RNP technology. Functional assays showed that *RSPRY1* KO fibroblasts exhibited increased wound closure, a phenotype that was abrogated in the double KO cells. These findings represent the first direct evidence linking RSPRY1 deficiency to overactivation of TGF-β signaling, highlighting its role in the pathogenesis of SEMD. Furthermore, the observed relationship between RSPRY1 deficiency and TGF-β signaling might provide a foundation for novel targeted therapeutic strategies for patients with SEMD and other related skeletal dysplasias.

## 2. Results

### 2.1. Pathway Enrichment Analysis of DEGs

**Figure 1:**
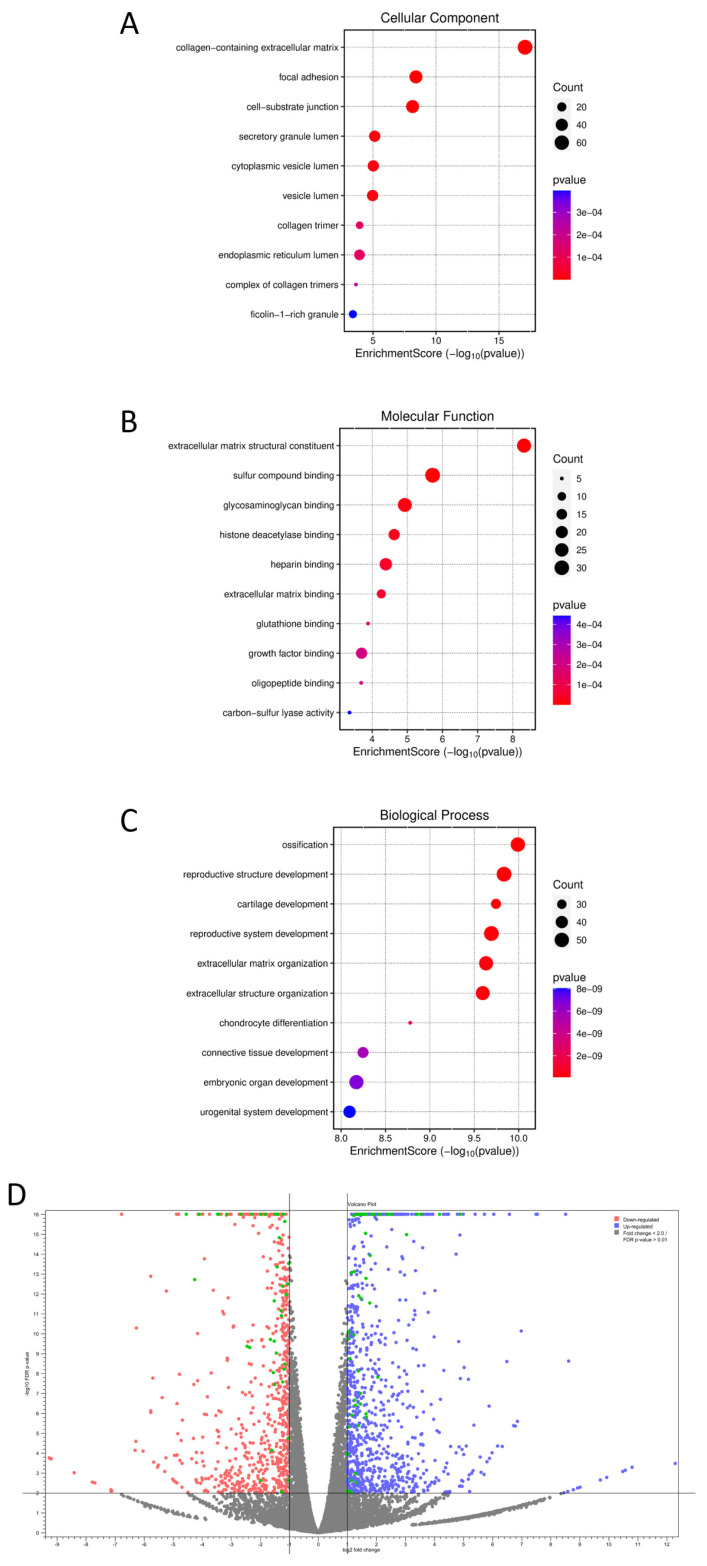
GO enrichment analysis and volcano plot of DEGs. This figure provides an overview of the GO enrichment and differential gene expression analysis of dermal fibroblasts with homozygous RSPRY1 mutations, as compared to CTRL fibroblasts. Each GO category, (**A**) Cellular Component (**B**) Molecular Function and (**C**) Biological Process, shows the enrichment score (−log10 of the *p* value) on the x-axis, with a dot size proportional to the number of genes contributing to the term and color intensity indicating statistical significance (*p* value). (**D**) The volcano plot shows the distribution of DEGs based on fold change (x-axis) and statistical significance (−log10 of the *p* value, y-axis). Red dots represent significantly upregulated genes. Blue dots represent significantly downregulated genes. Non-significant genes are shown in gray. Green dots represent the genes related to the TGF-β regulation of the ECM.

A comparison of dermal fibroblast samples from two SEMDFA patients with homozygous RSPRY1 mutations versus controls showed there were 456 genes with a decreased expression and 545 genes with an increased expression, an FDR *p* value ≤ 0.001, and an absolute fold change ≥ 2 (Supplementary Table S1). DEGs were subjected to GO enrichment analysis in all three categories: CC, MF, and BPs (Figure 1A–C). Significant enrichment was observed in terms related to the ECM, such as collagen-containing ECM, local adhesion, and cell-substrate junction. These findings show there is a strong association between DEGs, and ECM organization and related cellular structures. Enrichment analysis showed there was significant glycosaminoglycan binding, sulfur compound binding, and growth factor binding, indicating the functional role of DEGs in extracellular signaling and molecular interactions. The most prominent BPs enriched included cartilage development, ECM organization, and chondrocyte differentiation, indicating the potential involvement of DEGs in tissue development and differentiation, mainly related to connective tissue and cartilage.

The pathway enrichment analysis of DEGs showed there were several significantly enriched pathways, with TGF-β regulation of the ECM pathway being the most prominent. This pathway exhibited the most substantial enrichment, with a *p* value of 1.12 × 10^−24^ (Table 1 and Figure 1D). Essential genes enriched in this pathway included *SMAD3*, *COL1A1*, *WISP1*, and *RUNX2*, highlighting the central role of TGF-β signaling in ECM regulation and remodeling.

Other notable enriched pathways included the BDNF signaling pathway, which includes such genes as *FGF2*, *ITF2*, and *CRABP2*, and the oncostatin M signaling pathway, which involves such genes as *CEBPB*, *CXCL1*, and *PLAUR*. The beta-1 integrin cell surface interactions pathway, which includes such genes as *ITGA1*, *ITGA2*, and *COL1A1*, emphasizes the importance of cell adhesion and extracellular matrix interactions.

The additional pathways that were enriched included the interleukin-1 regulation of ECM, involving the genes *SMAD3*, *IL1RAP*, and *PTGES*, and the EGFR1 pathway, highlighting genes such as *ACO7*, *MMP1*, and *SLC20A1*. Other significant findings were the enrichment of Wnt interactions in lipid metabolism and the immune response pathway (essential genes *IL32*, *GPNMB*, and *COL1A1*), the biosynthesis of unsaturated fatty acids (genes *FADS2* and *ACO7*), and the Fas signaling regulation of apoptosis (genes *IFITM3*, *COL8A1*, and *ITM2C*). These findings show there was widespread disruption of lipid metabolism, immune signaling, and apoptosis regulation. Overall, the pathway enrichment analysis showed that TGF-β signaling was at the forefront of the observed molecular alterations, supported by additional pathways that highlight interconnected roles in inflammation, ECM remodeling, and cellular signaling.

### 2.2. Regulatory Network of Transcription Factors in TGF-β Signaling

**Figure 2:**
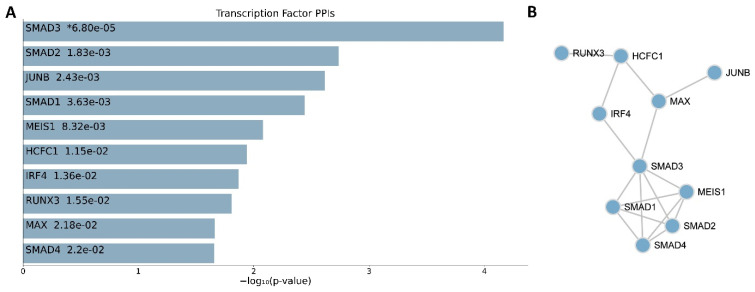
Enrichment and interaction analysis of TFs. TF enrichment and the interactions derived from the transcriptomic analysis of fibroblasts with RSPRY1 deficiency. (**A**) The bar chart shows the top enriched TFs based on their involvement in the DEGs. SMAD2 and SMAD3, both core components of the TGF-β signaling pathway, exhibited the highest enrichment, highlighting their dominant regulatory roles. The PPI network visualizes the interactions between the top enriched TFs. The asterisk indicates the most statistically significant TF. (**B**) The network shows the TFs’ collaborative regulatory activity in driving changes in gene expression, particularly within the TGF-β signaling pathway and ECM-related processes.

The observed significant enrichment of TGF-β signaling pathways highlights its central role in ECM regulation and suggests the involvement of key TFs orchestrating these changes in gene expression. The TFs that were enriched in the dataset were analyzed to investigate the associated regulatory mechanism, focusing on the TFs’ potential interactions and contributions to the molecular phenotype observed in RSPRY1-deficient fibroblasts. This analysis showed that SMAD2, SMAD3, and JUNB were the most significantly enriched TFs, highlighting their potential roles in regulating the changes in gene expression that were observed in fibroblast samples with homozygous *RSPRY1* mutations (Figure 2A). SMAD2 exhibited the highest enrichment, which indicated a dominant role in the TGF-β signaling pathway, the most significantly enriched pathway in the dataset. A PPI network analysis further underscored the central role of these TFs. SMAD2 and SMAD3 formed a tightly connected cluster with other key TFs, including JUNB, SMAD1, and MES1, which indicated there was coordinated regulatory activity. Other important TFs, such as RUNX3, IRF4, and HCFC1, were also integrated into the network, which highlighted their potential contributions to the transcriptional landscape (Figure 2B).

The above findings indicate that SMAD2 and SMAD3, as core components of the TGF-β signaling pathway, play a pivotal role in mediating the downstream effects of RSPRY1 deficiency. Their interactions with JUNB and other TFs highlight a complex regulatory network that governs ECM remodeling, inflammation, and other processes identified in the pathway enrichment analysis.

### 2.3. Phenotypic and Genetic Associations of RSPRY1 and the TGF-β Pathway

A detailed analysis was performed on the DEGs identified in the transcriptome data obtained from the fibroblasts of two patients carrying RSPRY1 homozygous mutations. These genes were then analyzed in conjunction with genes defined in the OMIM database and associated phenotypes. The findings showed that the genes in the DEGs list indicate clinical findings that phenotypically overlap with RSPRY1-related SEMD.

DEGs identified from the transcriptome data of fibroblasts from patients with homozygous RSPRY1 mutations were analyzed in the context of diseases that share clinical symptoms similar to those of SEMD. The aim was to understand the genetic factors that contribute to SEMD’s clinical manifestations. A detailed analysis was conducted by associating these DEGs with genes and phenotypes in the OMIM database. The findings showed that >16% of the DEGs were linked to diseases that exhibit clinical features similar to those observed in SEMD patients.

**Figure 3:**
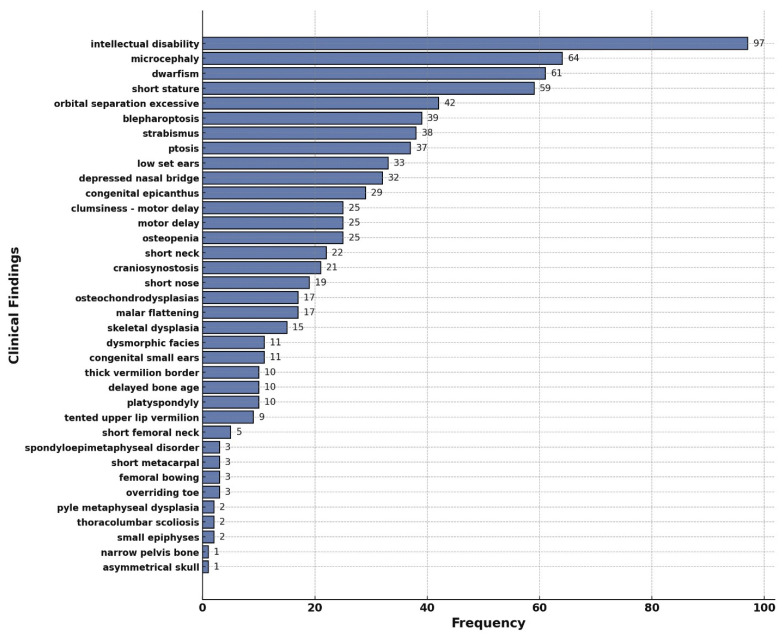
The frequency of clinical findings associated with RSPRY1 mutations. “Frequency” in this figure refers to the number of overlapping clinical findings associated with RSPRY1 mutations observed in the analyzed dataset. The DEGs list obtained from 2 patients carrying RSPRY1 mutations matched the genes defined in the OMIM database and related clinical phenotypes. Firstly, clinical findings phenotypically overlapping with RSPRY1 mutations were identified, and then a frequency analysis of these findings was performed. Clinical findings were grouped based on DisGenet terms, and the most common phenotypes were ranked. Each bar represents the number of times a specific phenotype was identified as overlapping with the genes linked to RSPRY1-associated pathways.

**Figure 4:**
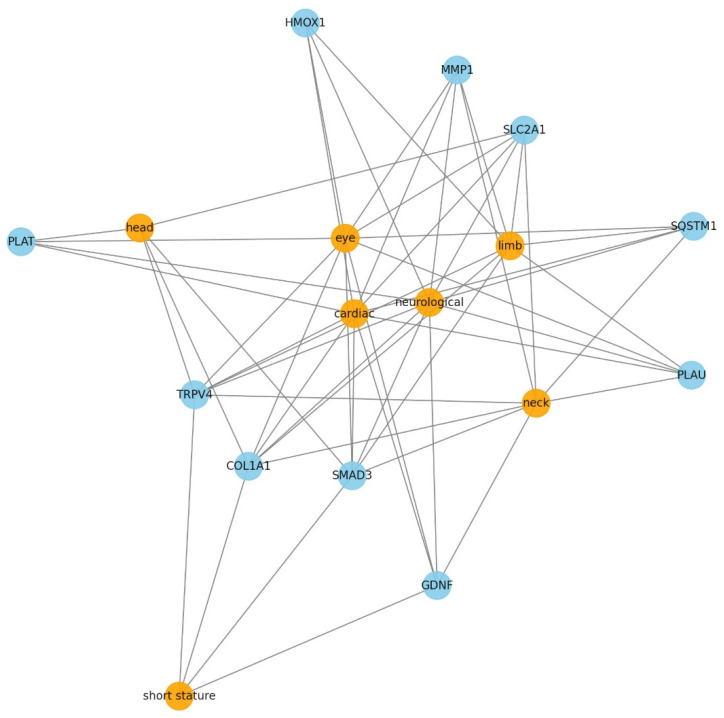
The top 10 genes and associated critical phenotypes network. This network visualization highlights the interactions between the top 10 genes with the highest association density and their linked phenotypic organ systems derived from analyzing RSPRY1-associated DEGs. Blue nodes represent the top 10 genes, including such key genes as SMAD3, COL1A1, and TRPV4, which are strongly associated with the TGF-β signaling pathway and ECM regulation. Orange nodes represent the phenotypic organ systems, including such critical features as eye, neck, short stature, and neurological phenotypes. Edges (lines) indicate the interaction or association between genes and phenotypic organ systems, with a denser connectivity representing a higher degree of association.

The bar chart in Figure 3 shows the number of DEGs that share similar phenotypes with the SEMD patients. The most commonly reported clinical symptoms were mental retardation (97 DEGs), microcephaly (64 DEGs), and dwarfism (61 DEGs), followed by short stature (59 DEGs) and increased orbital separation (42 DEGs). The genes associated with the clinical features shown in Figure 3 are provided in Supplementary Table S2. The 10 genes with the highest association density among the OMIM genes, and the phenotypic organ systems associated with these genes, were analyzed using the gene–phenotype network. Figure 4 shows the extent of the phenotypic effects of these genes and their interactions with each other. This analysis showed that the gene with the highest interaction density was *SMAD3*—a central node of the network with 462 interactions. This gene exhibited powerful associations with short stature, eye, and neurological phenotypes. Other vital genes included those involved in the TGF-β signaling pathway or associated with ECM regulation, such as *COL1A1*, *TRPV4*, and *GDNF*. To further investigate the role of RSPRY1 and its interaction with SMAD3 in cellular processes, we generated *RSPRY1* KO, *SMAD3* KO, and *RSPRY1* + *SMAD3* double KO fibroblasts.

### 2.4. Characterization of KO Fibroblasts

TGF-β superfamily members are involved in various cellular processes, such that alterations in the TGF-B signaling pathway can lead to changes in cell characteristics, including proliferation, migration, and apoptosis [12,15]. To assess the molecular and cellular characteristics of *RSPRY1* KO, *SMAD3* KO, and *RSPRY1* + *SMAD3* double KO fibroblasts, proliferation assays and beta-galactosidase staining for senescence were performed. Additionally, apoptosis was assessed for detecting healthy, early/late-apoptotic, and necrotic cell rates using flow cytometry (Annexin V-FITC and 7-AAD staining), which showed that there were not any significant apoptotic effects during the experimental conditions. Cell proliferation was measured via assessing the Ki-67 proliferation index and evaluating the doubling time using a real-time cell analyzer under standard culture conditions. There were not any significant differences observed between the KO groups (*RSPRY1* KO, *SMAD3* KO, and *RSPRY1* + *SMAD3* double KO) and the control fibroblasts (Cas9 only), which indicated that RSPRY1 and SMAD3 deficiencies did not affect cellular proliferation. Senescence was also evaluated using beta-galactosidase staining, and similar staining levels were observed across all groups, which indicated that neither RSPRY1 nor SMAD3 deficiency induced cellular senescence under the tested conditions (Supplementary Figures S1 and S2 and Supplementary Tables S3 and S4). Additionally, a qRT-PCR analysis was performed for selected genes identified as significantly altered in patient-derived RSPRY1 fibroblasts and assessed in the *RSPRY1* KO cells. The similar expression pattern of these genes observed in *RSPRY1* KO fibroblasts further highlights these models’ functional similarities (Supplemental Figure S3).

### 2.5. The Impact of RSPRY1 Deficiency on Cell Migration: Evidence of SMAD3 Dependence

**Figure 5:**
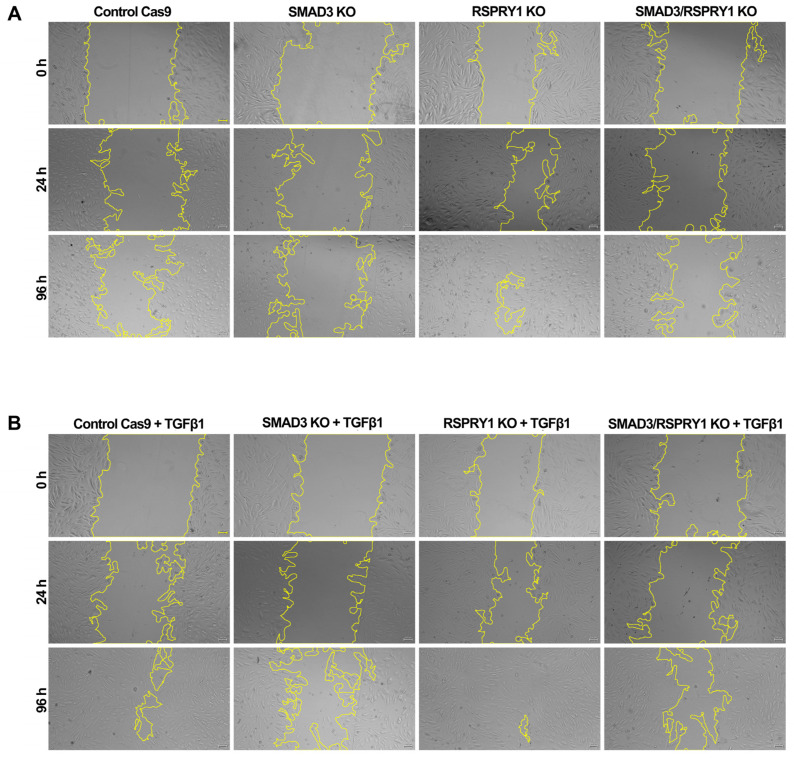
Wound-healing assay. Representative wound-healing assay images at 0, 24, 48, and 96 h. (**A**). Wound-healing dynamics in fibroblast groups, including CTRL (Cas9), RSPRY1 KO, SMAD3 KO, and RSPRY1 + SMAD3 + double KO cells without TGF-β supplementation, highlighting differences in wound closure rates across conditions. (**B**). Wound-healing dynamics in the same experimental fibroblast groups under TGF-β supplementation. Yellow outlines indicate wound borders at each time point. Scale bar, 100 μm.

**Figure 6:**
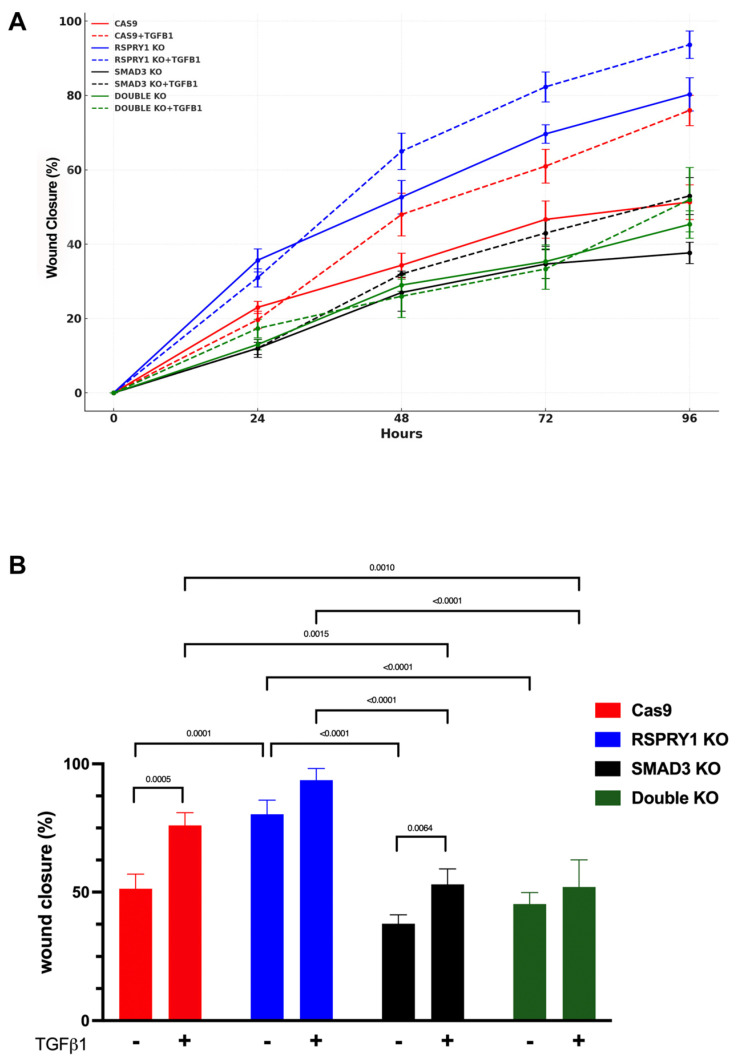
Wound closure percentage. (**A**). Line graph showing the percentage of wound closure over time (0–96 h), with error bars representing standard deviations. (**B**). Bar graph comparing wound closure percentages across fibroblast groups at 96 h under TGF-β-supplemented and standard conditions.

To further investigate the role of RSPRY1 and its interaction with SMAD3 in cellular processes, *RSPRY1* KO, *SMAD3* KO, and *RSPRY1* + *SMAD3* double KO fibroblasts were generated. Wound-healing assays were performed under two conditions: growth medium supplemented with 0.5% FBS and the same medium supplemented with recombinant TGF-β1 protein. *RSPRY1* KO fibroblasts exhibited a significant increase in wound closure ability, as compared to CTRL fibroblasts, which indicated that RSPRY1 deficiency enhanced cell motility (Figure 5 and Figure 6); however, this improved wound-healing capacity was not observed in the *RSPRY1* + *SMAD3* double KO fibroblasts, indicating that SMAD3 plays a critical role in mediating the effects of RSPRY1 loss on cell motility. These findings were consistent across both standard and TGF-β1-supplemented conditions, further highlighting the interplay between RSPRY1 and SMAD3 in TGF-beta-mediated pathways. Interestingly, TGF-β1 supplementation significantly accelerated wound closure in Cas9 CTRL cells, which highlighted a direct role in promoting cellular motility. This effect, however, was limited in *RSPRY1* KO and *RSPRY1* + *SMAD3* double KO cells, which suggested that endogenous TGF-β signaling might already be sufficient to drive cell motility in the absence of RSPRY1. The observation that *SMAD3* KO fibroblasts exhibited a lower wound closure rate than *RSPRY1* + *SMAD3* double KO fibroblasts under TGF-β1-free conditions suggests that RSPRY1 deficiency partially compensates for the absence of SMAD3 in cellular processes. This compensatory effect might be indicative of a distinct mechanism by which RSPRY1 interacts with other components of the TGF-β signaling pathway or alternative signaling cascades.

The significant increase in wound closure observed in *RSPRY1* KO fibroblasts, alongside the abrogation of this effect in the *RSPRY1* + *SMAD3* double KO fibroblasts, highlights the critical interplay between RSPRY1 and SMAD3 in TGF-β-mediated cellular processes. While the limited response to TGFB1 supplementation in *RSPRY1* KO and *RSPRY1* + *SMAD3* double KO cells is indicative of a possible constitutive activation of the TGF-β pathway in RSPRY1-deficient cells, the pronounced effect of TGF-β1 in Cas9 cells suggests that the pathway remains dynamic and responsive in the absence of *RSPRY1* mutations. Moreover, proliferation-independent effects were confirmed via Ki-67 staining, using flow cytometry and a doubling time analysis with the xCELLigence system, which showed there were not any significant differences in the proliferation rates across conditions. These findings indicate that RSPRY1 acts as a regulator within the TGF-β pathway, influencing such cellular dynamics as motility in a SMAD3-dependent manner.

## 3. Discussion

The present findings provide novel insights into the molecular mechanisms underlying RSPRY1 deficiency and its association with SEMD. Based on comprehensive transcriptomic, phenotypic, and functional analyses, the present findings show that RSPRY1 is a critical regulator of the TGF-β signaling pathway, a central axis in skeletal development and ECM dynamics. Moreover, they show how RSPRY1 deficiency disrupts ECM remodeling, cell motility, and cellular signaling processes, ultimately contributing to the SEMD phenotype.

The TGF-β superfamily is essential for intramembranous and endochondral ossification during skeletal development. The TGF-β pathway is tightly regulated by chondrocytes in the growth plate for maintaining bone and cartilage health [12]. Disruptions in this pathway often lead to defective chondrogenesis and impaired ossification, contributing to skeletal dysplasia and reduced height. Faden et al. reported that RSPRY1 is highly expressed in developing endochondral bones, particularly during primary ossification in mouse models, highlighting its potential role in skeletal development [4]. Consistent with these findings, the present study’s transcriptomic analysis of RSPRY1-deficient fibroblasts shows there is significant dysregulation of ossification-related genes, including *SMAD1*, *SMAD3*, *TGFB3*, and *NOG*, all of which are key regulators of TGF-β signaling. The altered expression of these genes suggests that RSPRY1 deficiency might impair TGF-β-driven pathways essential for proper endochondral ossification and bone homeostasis. These findings further underscore the critical role of RSPRY1 as a potential upstream regulator within the TGF-β signaling pathway, and the link between its loss of function and skeletal abnormalities.

The most striking finding of the present study is the significant enrichment of the TGF-β regulation of the ECM pathway in fibroblasts from the two patients with homozygous *RSPRY1* mutations. Key TFs, including SMAD2 and SMAD3, were observed to be pivotal regulators that form a tightly interconnected PPI network. This finding highlights the dominant role of TGF-β signaling in mediating the downstream effects of RSPRY1 deficiency. Overactivation of this pathway likely disrupts ECM homeostasis by driving the aberrant production of ECM proteins, consistent with clinical manifestations of SEMD, which is characterized by progressive epiphyseal and metaphyseal deformities and impaired skeletal homeostasis [16,17,18,19].

Functional wound-healing assays in the present study confirmed the critical role of RSPRY1 in cellular dynamics. *RSPRY1* KO fibroblasts exhibited an enhanced wound closure ability, suggesting increased cell motility; however, this phenotype was abrogated in *RSPRY1* + *SMAD3* double KO fibroblasts, indicating SMAD3′s essential role in mediating the effects of RSPRY1 deficiency. Interestingly, TGF-β1 supplementation significantly accelerated wound closure in Cas9 CTRL fibroblasts, highlighting the direct role of TGF-β in promoting cellular motility. In contrast, TGF-β1 had limited effects in *RSPRY1* KO and *RSPRY1* + *SMAD3* double KO fibroblasts, suggesting that endogenous TGF-β signaling might already be constitutively active in RSPRY1-deficient cells. This constitutive activation might explain the reduced responsiveness to exogenous TGF-β1 and underscores RSPRY1′s regulatory role within the TGF-β pathway. It may also contribute to the progressive clinical symptoms observed in patients with SEMD.

The present study’s analysis also identified a broader dysregulation of ECM-related and inflammatory pathways in RSPRY1-deficient fibroblasts, including the interleukin-1 regulation of ECM, beta-1 integrin interactions, and the EGFR1 signaling pathway. These pathways are intricately linked to TGF-β signaling, suggesting there is a systemic impact of RSPRY1 deficiency on cellular homeostasis. Additional pathways, such as the biosynthesis of unsaturated fatty acids and the regulation of apoptosis, highlight the diverse cellular processes affected by *RSPRY1* mutations and their potential contribution to the complex SEMD phenotype.

BioGRID database analyses revealed that RSPRY1 has many ubiquitin ligases among its interaction partners. In particular, the fact that it carries the RING domain strengthens the hypothesis that RSPRY1 may indirectly regulate the TGF-β signaling pathway by ubiquitinating specific proteins in the extracellular environment. Given the impact of ubiquitination on protein stability and functions, this mechanism may be a critical focus in understanding the role of RSPRY1 in the TGF-β signaling pathway. In this context, interaction partners associated with RSPRY1 and the TGF-β signaling pathway, such as ADAMTSL1, stand out as potential targets for future studies [20]. The fact that ADAMTSL1 is associated with TGF-β suggests that this protein is involved in ECM regulation and TGF-β activation. Identifying the possible ubiquitination targets of RSPRY1 in this context and detailing the effects of these targets in the TGF-β signaling pathway may provide important information to elucidate the functional mechanism of RSPRY1 and develop therapeutic strategies for SEMD and other related pathologies. 

The phenotypic and genetic association analysis performed in the present study provided further insights into the clinical implications of RSPRY1 deficiency. The overlapping clinical findings associated with DEGs and OMIM phenotypes suggest that *RSPRY1* mutations broadly affect skeletal and extra-skeletal systems. The identification of *SMAD3* as the most highly interconnected gene in the phenotype–genotype network emphasizes its central role in TGF-β signaling and the SEMD phenotype.

These findings collectively reinforce the hypothesis that RSPRY1 deficiency disrupts the balance of TGF-β signaling, driving the molecular and cellular dysfunctions underlying SEMD; however, some limitations must be acknowledged when interpreting these findings. The present study focused on fibroblast cell models derived from two patients with homozygous *RSPRY1* mutations, which, while informative, might only partially capture the effects of RSPRY1 deficiency in other cell types or tissues. SEMD is a systemic disorder affecting multiple organ systems, including the skeletal, ocular, and possibly neurological systems; therefore, subsequent research should investigate the role of RSPRY1 in a broader range of cell types and tissues in order to more fully elucidate its systemic impact. Moreover, while the present findings highlight the molecular and cellular mechanisms underlying RSPRY1 deficiency, the clinical relevance of these findings requires validation via more extensive clinical studies. Evaluating the systemic effects of *RSPRY1* mutations in larger cohorts of SEMD patients is essential for establishing the broader phenotypic spectrum and for identifying potential therapeutic targets. While the present findings indicate that there is a mechanistic link between RSPRY1 deficiency and TGF-β signaling overactivation, it also paves the way for additional research—specifically, in vivo studies to validate these findings and investigate the therapeutic potential of modulating RSPRY1-related signaling pathways.

The narrow patient cohort due to the nature of this rare genetic disease is a limitation of our study. However, to test the findings from patient cells in a more comprehensive mechanistic framework, *RSPRY1* KO cell models were developed, and proof-of-concept experiments were performed on KO cells. KO cell models provided an important tool to understand the mechanistic basis of the findings in patient cells and to study the cellular effects of RSPRY1 in detail. However, further studies using a larger cohort of patients will be able to increase the accuracy and scope of these findings. In this context, we emphasize that the results of our study provide an important starting point towards understanding the molecular mechanisms of SEMD.

In conclusion, the present findings show that RSPRY1 is a critical regulator of ECM dynamics and skeletal homeostasis. By highlighting the interplay between RSPRY1 and TGF-β1 signaling, the present study provides a foundation for developing targeted therapies for patients with SEMD and other related skeletal dysplasias.

## 4. Materials and Methods

### 4.1. Patients and Human Primary Cell Cultures

The study included 2 patients that were diagnosed with SEMDFA at Hacettepe University, School of Medicine, Department of Pediatrics, Pediatric Genetics Division, Ankara, Türkiye. The diagnoses were based on our previous study [5]. Both patients carried a homozygous [c.377delT] [p.Ile126fs*] frameshift mutation in exon 2 of the *RSPRY1* gene (NM_133368). Dermal fibroblasts were obtained from the patients via skin biopsy. Control (CTRL) cells comprised of human dermal fibroblasts were obtained from healthy individuals, which were purchased from Gibco (Thermo Fisher Scientific, Waltham, MA, USA) and the American Type Culture Collection (ATCC, Manassas, VA, USA), respectively. Dermal fibroblasts were cultured in standard growth medium (DMEM-LG, Thermo Fisher Scientific, Waltham, MA, USA) supplemented with 10% FBS (Gibco, Thermo Fisher Scientific, Waltham, MA, USA), 1% Pen-Strep (Capricorn Scientific GmbH, Ebsdorfergrund, Germany), and 1% L-glutamine (Capricorn Scientific GmbH, Ebsdorfergrund, Germany). Next, they were incubated at 37 °C under 5% CO_2_ conditions. Cells at passage 3 were used for further experiments.

The study protocol was approved by the Hacettepe University Ethics Committee (GO 20/997) and was conducted in accordance with the Declaration of Helsinki. Written informed consent was obtained from the patients or their legal guardians.

### 4.2. RNA Sequencing and Data Analysis

RNA was isolated using the Single-Cell RNA Purification Kit (cat. no. 51800, Norgen Biotek Corp., Thorold, ON, Canada), according to the manufacturer’s instructions. The quality and quantity of RNA were measured spectrophotometrically using a NanoDrop 2000 (Thermo Fisher Scientific, Waltham, MA, USA). Library preparation was performed using the SENSE mRNA-Seq Library Prep Kit (cat no. 001.24, Lexogen, Wien, Austria), and the libraries were sequenced in duplicate using an Ion Proton Semiconductor Sequencer (Thermo Fisher Scientific, Waltham, MA, USA). Raw sequencing reads were imported into QIAGEN CLC Genomics Workbench v.23.0.5). The reads were aligned to the reference genome (hg19) using the RNA-Seq analysis tool and the following parameters: mismatch allowance = 2; maximum number of hits = 10; length and similarity fraction = 0.8. Gene expression levels were quantified using total count values, and the RPKM (reads per kilobase of transcript per million) and TPM (transcripts per million) were calculated for each gene. Differential expression analysis was performed using TMM (trimmed mean of M values)-based library normalization and the EdgeR algorithm in CLC Genomic Workbench. Genes with a fold change (FC)  > 2 and an adjusted *p* value  < 0.01 were considered differentially expressed genes (DEGs). Data were visualized via an expression volcano plot generated via CLC Genomic Workbench. DEGs were then subjected to functional enrichment analysis using the web-based tool Enrichr (https://maayanlab.cloud/Enrichr/ (accessed on 18 December 2024) [21]. Enrichr was used to compute statistical enrichment scores based on the over-representation analysis (ORA) method, and the results, highlighting top-ranked terms and pathways, were visualized via bar graphs and interpreted in the study context. Gene ontology (GO) analysis for biological processes (BPs), cellular components (CCs), and molecular function (MF) were conducted with clusterProfiler and pathview [22,23].

### 4.3. Genome Editing in Human Primary Cells

Genome editing was performed using CRISPR-Cas9 ribonucleoprotein (RNP) complexes in a single-step workflow optimized for primary human fibroblasts [24]. Guide RNAs (gRNAs) consisting of crRNA and tracrRNA were purchased from Integrated DNA Technologies (IDT, Coralville, IA, USA). The crRNAs for the RSPRY1 gene were designed by identifying target regions shared across the CHOPCHOP [25], CRISPOR [26], and IDT online CRISPR design tool outputs. This approach ensured the selection of a high-confidence target region with optimal on-target efficiency and minimal off-target effects. Each crRNA sequence targeted the coding region of the gene, carefully avoiding known polymorphisms and predicted secondary structures that could interfere with Cas9 binding. A crRNA with established efficacy, previously validated by Martufi et al., was selected for the *SMAD3* gene to ensure reliable gene targeting [24]. The crRNA and tracrRNA were mixed at equimolar concentrations and annealed via heating at 95 °C for 5 min, and then were cooled to room temperature to form functional gRNA duplexes.

The RNP complex was formed by incubating the annealed gRNA with Cas9 nuclease (Alt-R S.p. Cas9 Nuclease V3, IDT, Coralville, IA, USA) at the ratio of 1.2:1. To enhance delivery efficiency, an electroporation enhancer (Alt-R Electroporation Enhancer, IDT, Coralville, IA, USA) was included in the mixture. Primary fibroblasts at approximately 80% confluency were trypsinized, washed with phosphate-buffered saline (PBS) to remove RNase activity, and then resuspended in P3 buffer (Lonza, Basel, Switzerland). Electroporation was performed using the 4D-Nucleofector System (Lonza, Basel, Switzerland) and the CM-138 program. Each reaction contained 250,000 cells in a total volume of 20 µL. Following electroporation, cells were transferred to culture media supplemented with 10% fetal bovine serum (FBS) (Gibco, Thermo Fisher Scientific, Waltham, MA, USA) and incubated at 37 °C with 5% CO_2_. Editing efficiency was assessed via targeted deep sequencing using the MiSeq Sequencing System (Illumina, San Diego, CA, USA), achieving >90% KO efficiency without antibiotic selection (Supplementary Figure S4).

### 4.4. Quantitative RT-PCR

Cells were washed with PBS solution, and total RNA was isolated using the Norgen Total RNA Isolation Kit (Norgen Biotek Corp., Thorold, ON, Canada), according to the manufacturer’s instructions. The quality and concentration of RNA were assessed spectrophotometrically using a NanoDrop 2000 spectrophotometer (Thermo Fisher Scientific, Waltham, MA, USA). Complementary DNA (cDNA) was synthesized from 500 ng of total RNA using the QuantiTect Reverse Transcription Kit (Qiagen, Hilden, Germany), according to the manufacturer’s instructions. The quantitative real-time PCR (qRT-PCR) was performed using LightCycler^®^ 480 SYBR Green Master Mix (Roche Diagnostics, Basel, Switzerland) and a LightCycler^®^ 480 System (Roche Diagnostics, Basel, Switzerland). All reactions were performed in triplicate to ensure reproducibility, and amplification conditions were set according to the manufacturer’s instructions. The primer sequences are given in Supplementary Table S5.

### 4.5. Flow Cytometry and Real-Time Monitoring of Proliferation

A comprehensive flow cytometric analysis of Annexin V/7-AAD, senescence-associated β-galactosidase (SA-β-Gal), and Ki-67 was conducted to evaluate apoptosis, cellular senescence, and proliferation in the CTRL and KO dermal fibroblast cells. Apoptosis was studied using an FITC Annexin V Apoptosis Detection Kit (cat. no. 640914, BioLegend, San Diego, CA, USA). Staining was performed using Cell Staining Buffer (cat. no. 420201, BioLegend, San Diego, CA, USA), Annexin V-FITC (cat. no. 640906, BioLegend, San Diego, CA, USA), and 7-AAD viability dye PerCp (Ref: IM3630C, Beckman Coulter Inc., Brea, CA, USA). SA-β-Gal activity, a widely recognized marker of cellular senescence, was detected using a fluorescence-based substrate (cat. no. ab228562, Abcam, Cambridge, UK). The cells were incubated for 90 min at 5% CO_2_ and 37 °C, then washed with the 1X wash buffer provided with the kit and trypsinized. For the intracellular staining of the proliferation marker Ki-67, cells were treated with Anti-Ki67-APC (cat. No. 350513 BioLegend, San Diego, CA, USA). Fixation and permeabilization were conducted using a fixation buffer (Ref: TNB-B222-L100, Tonbo Biosciences, San Diego, CA, USA) and permeabilization buffer (Ref: TNB-1213-L150, Tonbo Biosciences, San Diego, CA, USA), respectively. All subsequent analyses were performed using a NovoCyte flow cytometer (ACEA Biosciences, San Diego, CA, USA).

Cell proliferation was monitored in real time using the xCELLigence DP system (Agilent Technologies Inc., Santa Clara, CA, USA). Electrical impedance was measured using gold microelectrodes embedded in the bottom of a single-use 16-well plate, and real-time changes during proliferation were recorded. First, the background impedance of the empty growth media was measured for 30 min. Then, fibroblast cells from each group were seeded into the wells, and cell proliferation was monitored every 15 min for 96 h. Between 50 and 96 h, the cells’ doubling times (DTs) were calculated using RTCA software v2.0.0.1301.

### 4.6. Wound-Healing Assay

Scratch assays of dermal fibroblasts were used to assess wound-healing capacity. Cells (Cas9 CTRL, *RSPRY1* KO, *SMAD3* KO, and *RSPRY1* + *SMAD3* double KO) were cultured in 24-well tissue plates at a density of 70,000 cells well^−1^ in DMEM medium supplemented with 10% FBS until 90% confluence was achieved. Next, a linear scratch was created in the center of each well using a 1000 µL pipette tip, and the wells were washed with PBS to remove cellular debris. After scratch creation, the medium was replaced with (i) fresh 0.5% serum-supplemented culture medium to reduce the effect of proliferation or (ii) fresh 0.5% serum and 10 ng mL^−1^ human TGF-β1 recombinant protein (Cell Signaling Technology, Beverly, MA, USA) supplemented culture medium. The initial wound width and area were imaged using a Leica Dmi1 inverted microscope (Leica, Berlin, Germany) at the 0 h time point. Following the wounding process, cells were incubated for 24, 48, 72, and 96 h, and images were obtained at specified intervals to evaluate the rate of wound closure. The wound area at each time point was quantified relative to the initial wound area, providing a quantitative assessment of cell migration. Image analysis was performed using an ImageJ plugin for Fiji software v.2.14.0/1.54f [27]. The percentage of wound closure was calculated using the following equation:

Wound Closure (%) = [(A_t = 0 − A_t = Δt)/A_t = 0] × 100

where A_t = 0 represents the initial wound area, and A_t = Δt represents the wound area after n hours from the initial scratch (both in µm^2^).

### 4.7. Statistics and Data Visualization

Data were analyzed using GraphPad Prism v.9.0. All experiments were performed in 3 independent replicates, and statistical analysis was performed using a one-way or two-way analysis of variance (ANOVA), as applicable, followed by Tukey’s post hoc test for multiple comparisons. The level of statistical significance was set at *p* < 0.05. Network analysis and graphs were visualized using Python and relevant libraries, including Pandas, NumPy, and SciPy, for data processing and statistical testing. Data visualization was performed using Matplotlib and Seaborn, which generated publication-quality plots, including bar charts, line graphs, and scatter plots. Cytoscape was employed to construct and analyze PPI networks and phenotype association graphs. Specific figures, such as the wound-healing dynamics and transcription factor (TF) interaction networks, were created using Matplotlib and Cytoscape integration.

## Figures and Tables

**Table 1:** Pathway enrichment analysis of DEGs. The table summarizes the top pathways significantly enriched based on the transcriptomic analysis of fibroblasts with homozygous RSPRY1 mutations. Each pathway is listed alongside its *p* value, *q* value (false discovery rate-adjusted *p* value), and the overlapping genes contributing to the enrichment. The enrichment of these pathways, particularly the prominence of TGF-beta signaling, underscores the systemic and multifactorial impact of RSPRY1 deficiency on cellular and molecular mechanisms, contributing to the observed phenotype.

Term	*p* Value	Genes
TGF-beta regulation of extracellular matrix	1.12 × 10^−24^	*DIRAS3*; *CNTNAP1*; *OXTR*; *NRP2*; *CITED2*; *SUV39H1*; *CCNF*; *TNC*; *SLC2A1*; *NUDT2*; *CXCL1*; *SQRDL*; *SLC7A11*; *WISP1*; *IFIT3*; *WISP2*; *EFEMP1*; *DPYSL2*; *RUVBL2*; *CTSK*; *PIM1*; *AOX1*; *JUNB*; *FADS1*; *CTSC*; *DACT1*; *POSTN*; *HMGCS1*; *MME*; *F2R*; *ABCC9*; *ACSL3*; *AXIN2*; *SREBF2*; *MTHFD2*; *HAND2*; *PPAP2B*; *GAS1*; *TRIB3*; *PRKD1*; *LPHN2*; *CFD*; *COLEC12*; *CRABP2*; *CEBPD*; *STX17*; *SLC20A1*; *MGST2*; *NEDD9*; *LTBP2*; *SLC1A4*; *AEBP1*; *PTGS2*; *PTGS1*; *SELENBP1*; *DPP4*; *SRPX2*; *GPNMB*; *HAS2*; *SLIT3*; *GBP2*; *CA12*; *ARNT2*; *SVIL*; *TCF7L1*; *SMAD3*; *ANGPT1*; *LUM*; *AKR1C1*; *TFPI2*; *AKR1C3*; *AKR1C2*; *NR2F2*; *PTPN13*; *AGT*; *EPOR*; *VEGFA*; *COL1A1*; *FOSL1*; *MFAP5*; *UCK2*; *BAMBI*; *SCD*; *CTH*; *QPCT*; *COL7A1*; *ITGA11*; *PIR*; *ID4*; *SNAI2*; *CRLF1*; *PFKP*; *BCL2L1*
BDNF signaling pathway	9.16 × 10^−10^	*BTG2*; *IFITM2*; *CRABP2*; *MAST4*; *ADM*; *PLAT*; *PTGS2*; *CLU*; *PTGS1*; *STIP1*; *NUAK1*; *SLIT3*; *FLNC*; *PRSS3*; *JUNB*; *FADS1*; *ZKSCAN1*; *DUSP5*; *HSPA8*; *JUN*; *GCH1*; *GDF15*; *AKR1C1*; *ITGA2*; *TFPI2*; *HPCAL1*; *AKR1C3*; *VEGFA*; *CENPF*; *GAP43*; *GAL*; *CDH11*; *TXNIP*; *BIRC5*; *CSPG4*; *TRIB2*; *HSPA1B*; *VCL*; *HSPA1A*
Oncostatin M	1.46 × 10^−8^	*FLG*; *FBN2*; *CRABP2*; *CEBPD*; *TNC*; *PLAT*; *CXCL1*; *PRDM1*; *PTGS2*; *HIF1A*; *AURKA*; *ADAMTS4*; *SELENBP1*; *MT2A*; *NUAK1*; *PLAU*; *HMOX1*; *CCL2*; *SNCG*; *JUNB*; *TGM2*; *ZBTB18*; *JUN*; *SERPINB1*; *SERPINB2*; *HMGCS1*; *MMP1*; *AKR1C1*; *F2R*; *AKR1C3*; *ACSL3*; *AMPD3*; *PGF*; *FOSL2*; *VEGFA*; *COL1A1*; *CH25H*; *KRT18*; *CDH11*; *IL6ST*; *CRLF1*
Beta-1 integrin cell surface interactions	1.11 × 10^−7^	*COL18A1*; *ITGA2*; *COL11A1*; *ITGA1*; *TNC*; *VEGFA*; *COL1A1*; *COL3A1*; *MDK*; *PLAU*; *COL7A1*; *ITGA11*; *ITGA8*; *ITGA6*; *CSPG4*; *TGM2*
Interleukin-1 regulation of extracellular matrix	4.93 × 10^−7^	*SRGN*; *IL15RA*; *SMAD1*; *JUN*; *SMAD3*; *SERPINB2*; *MMP1*; *STMN2*; *CXCL1*; *NR2F2*; *AMPD3*; *PTGS2*; *MMP10*; *COMP*; *FOSL1*; *MT2A*; *ALDH1A1*; *CCL2*; *PTX3*; *PIM2*; *PTGES*
EGFR1 pathway	5.55 × 10^−7^	*ACOT7*; *MME*; *MMP1*; *SLC20A1*; *ITGA2*; *MT1M*; *HPCAL1*; *PKMYT1*; *VEGFA*; *SELENBP1*; *PNP*; *TCOF1*; *PLAU*; *PPAP2B*; *MT1F*; *SLC16A7*; *TXNIP*; *HAS2*; *SPRY2*; *FKBP4*; *GABRE*; *SQSTM1*; *TRIB2*; *VCL*
Wnt interactions in lipid metabolism and immune response	9.88 × 10^−6^	*IL32*; *GPNMB*; *FZD4*; *MMP1*; *COL8A1*; *CXCL1*; *HES1*; *TCF4*; *AXIN2*; *TEK*; *WISP1*
Biosynthesis of unsaturated fatty acids	4.82 × 10^−5^	*FADS2*; *ACOT7*; *SCD*; *ELOVL2*; *SCD5*; *ACOT1*; *FADS1*
Gastrin pathway	4.94 × 10^−5^	*FOSL1*; *JUN*; *SERPINB2*; *TNC*; *HES1*; *PTGS2*; *KLF4*; *SREBF2*; *MYLK*; *VEGFA*
FSH regulation of apoptosis	1.43 × 10^−4^	*IFITM3*; *COL18A1*; *IFITM1*; *IFITM2*; *SDC2*; *PTPRN*; *MSMO1*; *CLU*; *RASSF2*; *SLC16A7*; *FLNC*; *PTGDS*; *GBP1*; *ANGPT1*; *BDNF*; *TFPI2*; *RAB27A*; *RANGAP1*; *VEGFA*; *CH25H*; *DAB2*; *KRT18*; *RAB31*; *ID4*; *CPE*; *PRKD1*; *DHCR7*; *VCL*

## Data Availability

Data is contained within the article and Supplementary Materials.

## Supplementary Materials

The following supporting information can be downloaded at: https://www.mdpi.com/article/10.3390/ijms26031134/s1.
