# Supplementary material for: Unraveling the Role of RSPRY1 in TGF-β Pathway Dysregulation: Insights into the Pathogenesis of Spondyloepimetaphyseal Dysplasia
Source: Int J Mol Sci. 2025 Jan 28;26(3):1134. doi: 10.3390/ijms26031134 (PMC11817781; doi:10.3390/ijms26031134)
Supplement: Supplementary file 1 [file ijms-26-01134-s001.zip › ijms-3410596-supplementary/Revision_Supp data all/Supp data_marked.pdf]

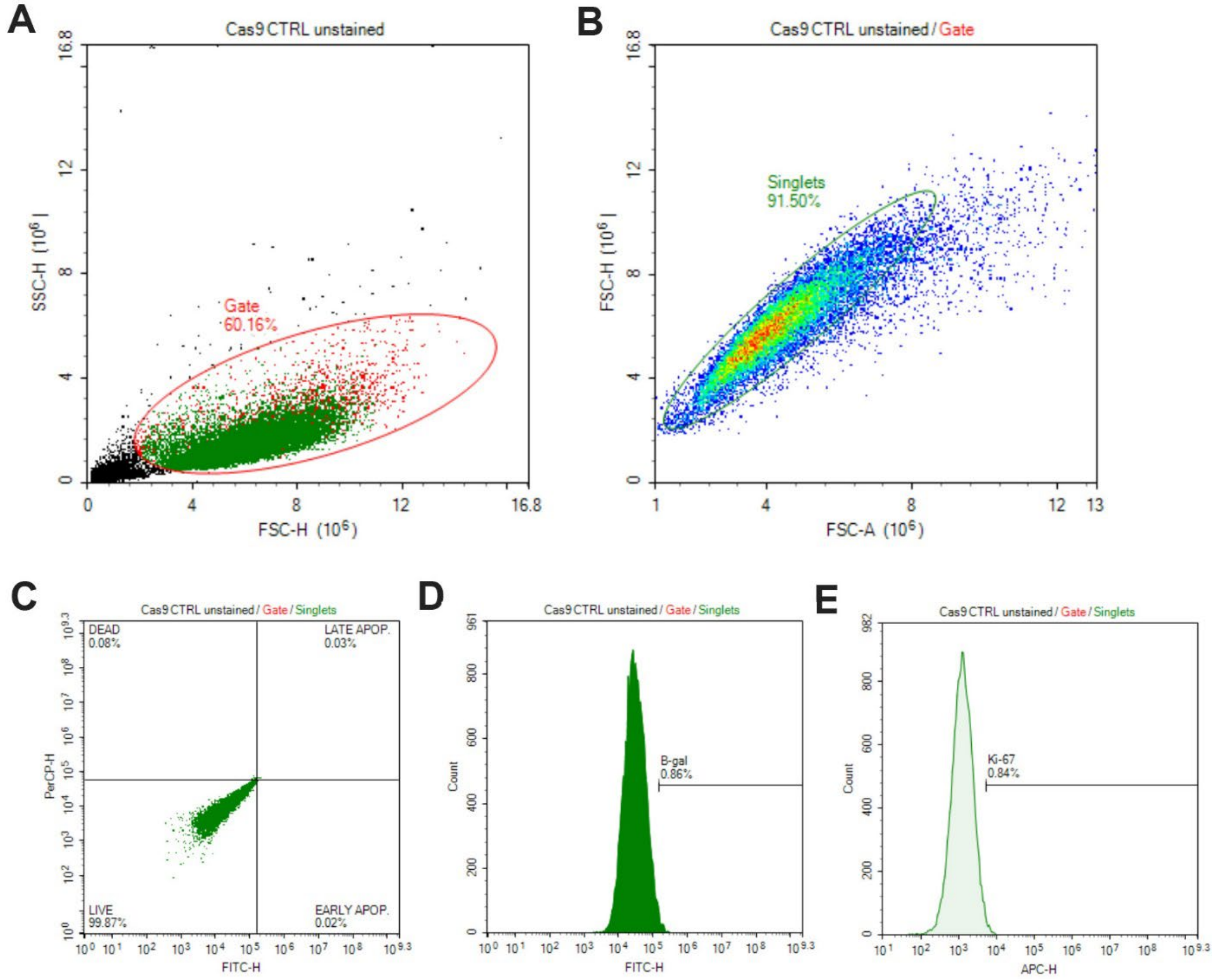


**Figure S1.** The gating strategy for the flow cytometry analysis. (**A**) Cells were initially gated based on forward scatter (FSC) versus side scatter (SSC) to identify the target cell population. (**B**) Then, gating was performed on FSC-Height (FSC-H) versus FSC-Area (FSC-A) to exclude debris and doublets and isolate single cells within the singlets gate. Finally, based on the assay, all subpopulations were assessed using (**C**) Annexin V-FITC versus 7-AAD PerCP scatter plot to distinguish live cells, as well as those undergoing early and late apoptosis, (**D**) B-gal FITC histogram plot for Senescence-associated β-galactosidase (SA-β-Gal) activity and (**E**) Ki-67 APC histogram plot for proliferation. Auto-fluorescence of each biological replicate was used as a negative control. An unstained Cas9 CTRL group was used for representation.

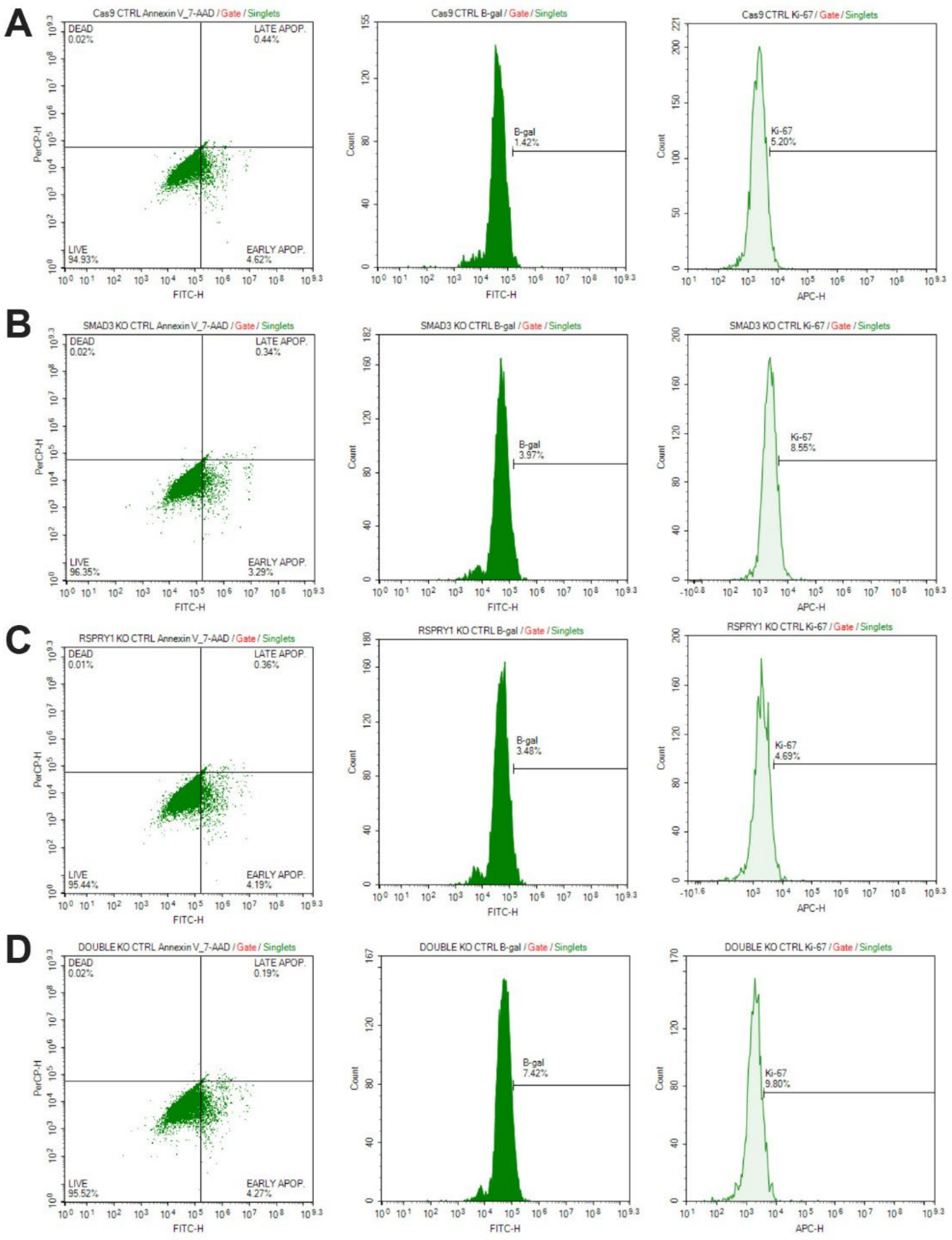


**Figure S2.** Flow cytometric analysis of apoptosis, senescence, and proliferation in primary human dermal fibroblast cells**.** Analysis of Annexin V-FITC and 7-AAD-PerCP staining was performed to classify cell populations into viable (Annexin V-/7-AAD-), necrotic (Annexin V-/7-AAD+), early apoptotic (Annexin V+/7-AAD-), and late apoptotic (Annexin V+/7-AAD+) cell (left panel). The middle and right panels show fluorescence histograms for senescence-associated β-galactosidase (SA-β-gal) (FITC channel) and Ki-67 (APC channel), respectively. Comparisons of Annexin V, 7-AAD, SA-β-gal, and Ki-67 staining were compared to unstained controls. Sample groups include: (**A**) Cas9 CTRL, (**B**) SMAD3 KO, (**C**) RSPRY1 KO, (**D**) Double KO.

**Table S3.** Percentage of gated populations from flow cytometric analysis of apoptosis, senescence, and proliferation in primary human dermal fibroblast cells.

| | **Annexin V/V_7-AAD** | | | | **β-gal** | **Ki-67** |
|---|---|---|---|---|---|---|
| | **Dead (%)** | **Late Apoptosis (%)** | **Early Apoptosis (%)** | **Live (%)** | **(%)** | **(%)** |
| **Cas9 Control** | 0.02 | 0.44 | 4.62 | 94.93 | 1.42 | 5.20 |
| **SMAD3 KO** | 0.02 | 0.34 | 3.29 | 96.35 | 3.97 | 8.55 |
| **RSPRY1 KO** | 0.01 | 0.36 | 4.19 | 95.44 | 3.48 | 4.69 |
| **Double KO** | 0.02 | 0.19 | 4.27 | 95.52 | 7.42 | 9.80 |

**Table S4.** Doubling Times (Hour) of edited and control fibroblast cells. Analysis is performed by using real-time impedance measurements with xCELLigence® instrument.

| | **Doubling Time(h)** | **SD** |
|---|---|---|
| **Control Cas9 Fibroblasts** | 30.53 | 0.26 |
| **SMAD3 KO Fibroblasts** | 31.03 | 0.31 |
| **RSPRY1 KO Fibroblasts** | 32.15 | 0.30 |
| **Double KO Fibroblasts** | 34.03 | 0.30 |

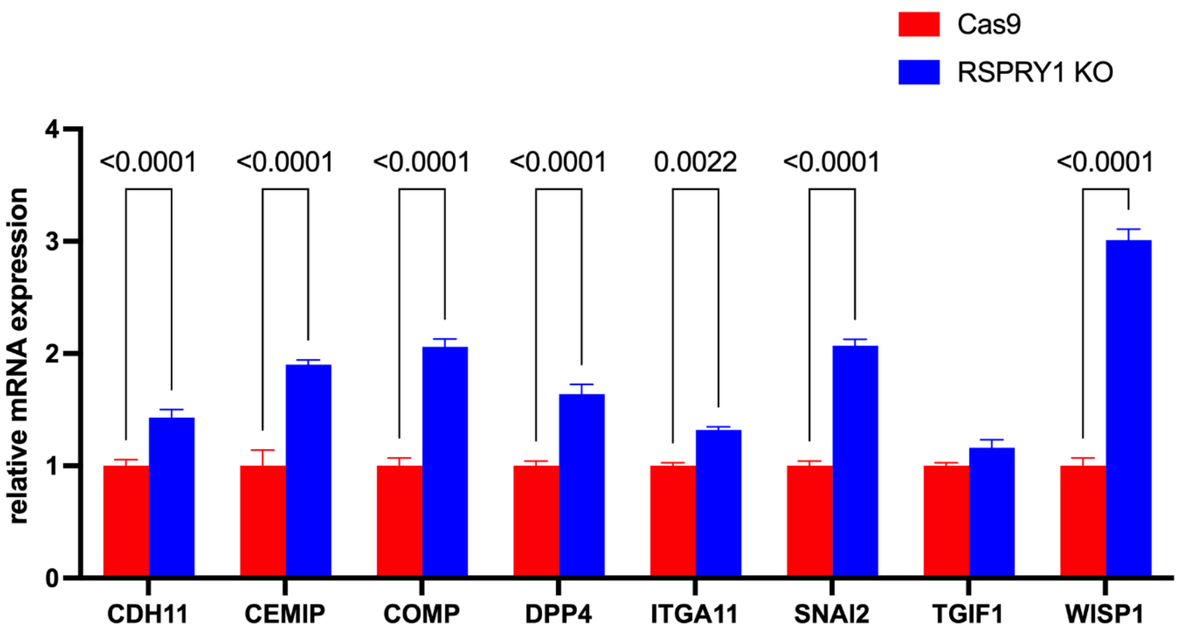


**Figure S3.** qRT-PCR analysis of selected genes in RSPRY1 KO fibroblasts. Bars represent the relative expression levels of key genes associated with TGF-β signaling and extracellular matrix regulation. Data are presented as mean ± standard deviation, normalized to a housekeeping gene (ACTB).

**Table S5.** Primer Sequences for Quantitative RT-PCR

| **Gene** | **Forward** | **Reverse** |
|---|---|---|
| ACTB (NM_001101.5) | CGCAAAGACCTGTACGCCAAC | GAGCCGCCGATCCACACG |
| CDH11 (NM_001797.4) | CCAGGACATTAATGACAACCC | GGTTGTCCTTCGAGGATACT |
| CEMIP (NM_001293298.2) | GGAGAGTTCCAAGCAGCA | CGTCAATCACCACCACCT |
| COMP (NM_000095.3) | AACAGTGCCCAGGAGGAC | TTGTCTACCACCTTGTCTGC |
| DPP4 (NM_001935.4) | AAAGGCACCTGGGAAGTCATCG | CAGCTCACAACTGAGGCATGTC |
| ITGA11 (NM_001004439.2) | GTTTCACCTCAACGACTACAG | CTTTGCTGGATCACCTTCTC |
| SNAI2 (NM_003068.5) | AGATGCATATTCGGACCCAC | AGATGAGCCCTCAGATTTGAC |
| TGIF1 (NM_003244.4) | CAGGCAAGAGAAGGAGAAGG | GCGTTGATGAACCAGTTACAG |
| WISP1 (NM_003882.4) | ACACTCATTAAGGCAGGGA | TTCCTACAGCTCAGGTTACAG |

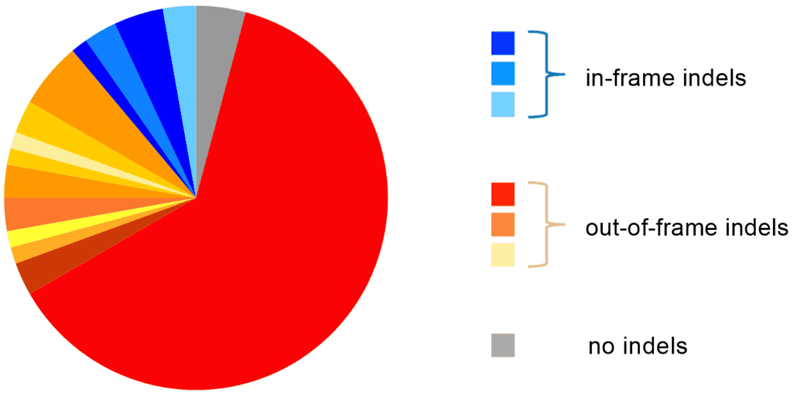


| Software | Name | sgRNA | PAM | bp | %GC | Exon | Strand |
|---|---|---|---|---|---|---|---|
| CHOPCHOP #2/ CRISPOR#1/IDT#1 | RSPRY1 gRNA | GGAGGTGCCGCTACTACCAT | GGG | 20 | 60 | 2 | + |

**Figure S4.** Sequence of the sgRNA and efficiency of genome editing. The pie chart represents the genomic landscape, with the colored segments indicating the type of mutations present. Shades of blue represent in-frame mutations, while shades of red indicate out-of-frame mutations. Gray segments correspond to regions without detectable indel mutations. The gradient of colors reflects the diversity within each mutation category, providing a visual representation of the distribution and types of genetic alterations within the genomic profile.
